# Assessing seroprevalence and associated risk factors for multiple infectious diseases in Sabah, Malaysia using serological multiplex bead assays

**DOI:** 10.3389/fpubh.2022.924316

**Published:** 2022-10-25

**Authors:** YuYen L. Chan, Catriona L. Patterson, Jeffrey W. Priest, Gillian Stresman, Timothy William, Tock H. Chua, Kevin Tetteh, Patrick Lammie, Chris Drakeley, Kimberly M. Fornace

**Affiliations:** ^1^Department of Infection Biology, London School of Hygiene and Tropical Medicine, London, United Kingdom; ^2^Division of Foodborne, Waterborne, and Environmental Diseases, United States Centers for Disease Control and Prevention, Atlanta, GA, United States; ^3^Infectious Diseases Society Sabah-Menzies School of Health Research Clinical Research Unit, Kota Kinabalu, Malaysia; ^4^Faculty of Medicine and Health Sciences, Universiti Malaysia Sabah, Kota Kinabalu, Malaysia; ^5^Division of Parasitic Diseases and Malaria, United States Centers for Disease Control and Prevention, Atlanta, GA, United States; ^6^Department of Disease Control, London School of Hygiene and Tropical Medicine, London, United Kingdom; ^7^School of Biodiversity, One Health and Veterinary Medicine, University of Glasgow, Glasgow, United Kingdom

**Keywords:** neglected tropical disease (NTD), serology, multiplex bead assay analysis, epidemiology - analytic (risk factors), Malaysia

## Abstract

**Background:**

Infectious diseases continue to burden populations in Malaysia, especially among rural communities where resources are limited and access to health care is difficult. Current epidemiological trends of several neglected tropical diseases in these populations are at present absent due to the lack of habitual and efficient surveillance. To date, various studies have explored the utility of serological multiplex beads to monitor numerous diseases simultaneously. We therefore applied this platform to assess population level exposure to six infectious diseases in Sabah, Malaysia. Furthermore, we concurrently investigated demographic and spatial risk factors that may be associated with exposure for each disease.

**Methods:**

This study was conducted in four districts of Northern Sabah in Malaysian Borneo, using an environmentally stratified, population-based cross-sectional serological survey targeted to determine risk factors for malaria. Samples were collected between September to December 2015, from 919 villages totaling 10,100 persons. IgG responses to twelve antigens of six diseases (lymphatic filariasis- Bm33, Bm14, BmR1, Wb123; strongyloides- NIE; toxoplasmosis-SAG2A; yaws- Rp17 and TmpA; trachoma- Pgp3, Ct694; and giardiasis- VSP3, VSP5) were measured using serological multiplex bead assays. Eight demographic risk factors and twelve environmental covariates were included in this study to better understand transmission in this community.

**Results:**

Seroprevalence of LF antigens included Bm33 (10.9%), Bm14+ BmR1 (3.5%), and Wb123 (1.7%). Seroprevalence of Strongyloides antigen NIE was 16.8%, for Toxoplasma antigen SAG2A was 29.9%, and Giardia antigens GVSP3 + GVSP5 was 23.2%. Seroprevalence estimates for yaws Rp17 was 4.91%, for TmpA was 4.81%, and for combined seropositivity to both antigens was 1.2%. Seroprevalence estimates for trachoma Pgp3 + Ct694 were 4.5%. Age was a significant risk factors consistent among all antigens assessed, while other risk factors varied among the different antigens. Spatial heterogeneity of seroprevalence was observed more prominently in lymphatic filariasis and toxoplasmosis.

**Conclusions:**

Multiplex bead assays can be used to assess serological responses to numerous pathogens simultaneously to support infectious disease surveillance in rural communities, especially where prevalences estimates are lacking for neglected tropical diseases. Demographic and spatial data collected alongside serosurveys can prove useful in identifying risk factors associated with exposure and geographic distribution of transmission.

## Introduction

Within the last decade, disease control efforts including mass drug administration, improved sanitation, and public health awareness have helped reduce the burden of neglected tropical (NTDs) and other infectious diseases in Malaysia. However, many of these diseases persist, especially among isolated, resource-constrained, and aboriginal communities in Sabah, resulting in sustained morbidity and chronic impact on quality of life (1). For example, helminth diseases in Malaysia include strongyloidiasis (2–4) and lymphatic filariasis (LF) (5) that can cause a range of illnesses leading to malnutrition and disability (6–8). Persistent protozoan diseases in Malaysia include giardiasis (9), toxoplasmosis (10), and malaria (11, 12). Giardiasis can result in malnutrition from chronic diarrhea (13, 14) while toxoplasmosis symptoms can vary from asymptomatic to severe clinical manifestations that occur typically in immunocompromised patients (15). In Malaysia, bacterial diseases include leptospirosis (16, 17), trachoma (18), and yaws (19) that can impact the skin, eyes, joints, and other parts of the body.

Current epidemiological trends are unknown for many of these infections due to the dearth of routine and reliable surveillance (18). Characterizing disease burden can be particularly difficult in low-transmission and post-elimination settings, especially if sub-clinical infections are common. Assessing cross-sectional population prevalence can help identify areas of transmission resurgence or introduction, but low transmission rates, mild morbidity, and limited resources may have reduced public health priority of systematic monitoring of these diseases. Since transmission of many of these pathogens geographically overlap and can result in co-infections, integrated, multi-disease monitoring would provide resource efficient alternatives compared with single disease surveillance (20). While diverse biological targets of tropical infections often require different laboratory methods to capture disease burden (e.g., stool microscopy, polymerase chain reaction, or antibody testing), a unified platform for monitoring exposure to diverse pathogens may help to overcome some of these logistical challenges toward concurrent NTD monitoring.

Integrated monitoring may be attainable using serological multiplex bead assays (MBA). MBAs can quantify immune responses to multiple pathogens from a single blood spot (21). Serology can effectively capture asymptomatic infections and reveal historical pathogen exposure by measuring pathogen-specific antibody responses (22). The use of serology in monitoring NTDs and vaccine preventable diseases (VPDs) has been applied in numerous settings (21–25). Furthermore, demographic and environmental data collected in population-based surveys provide key opportunities to assess potential and shared risk factors of the different diseases that may enhance controls strategies and community awareness. While certain socio-economic risk factors have been studied for several parasitic infections in Malaysia (4, 13, 26, 27), spatial and other risk factors are not well-characterized for many of these diseases.

To our knowledge, multiplex bead assays have yet to be applied to assessing NTD and parasitic disease seroprevalence and associated risk factors in Malaysia. In this study, we used MBA on samples collected during a 2015 cross-sectional survey in Northern Sabah, Malaysia to estimate population exposure to multiple pathogens. We aimed to (1) describe population level exposure to six infectious diseases, (2) assess pathogen-specific individual risk factors for exposure; and (3) determine spatial and environmental risk factors and predict population-level exposure probabilities.

## Methods

### Study site and sampling

**Figure 1:**
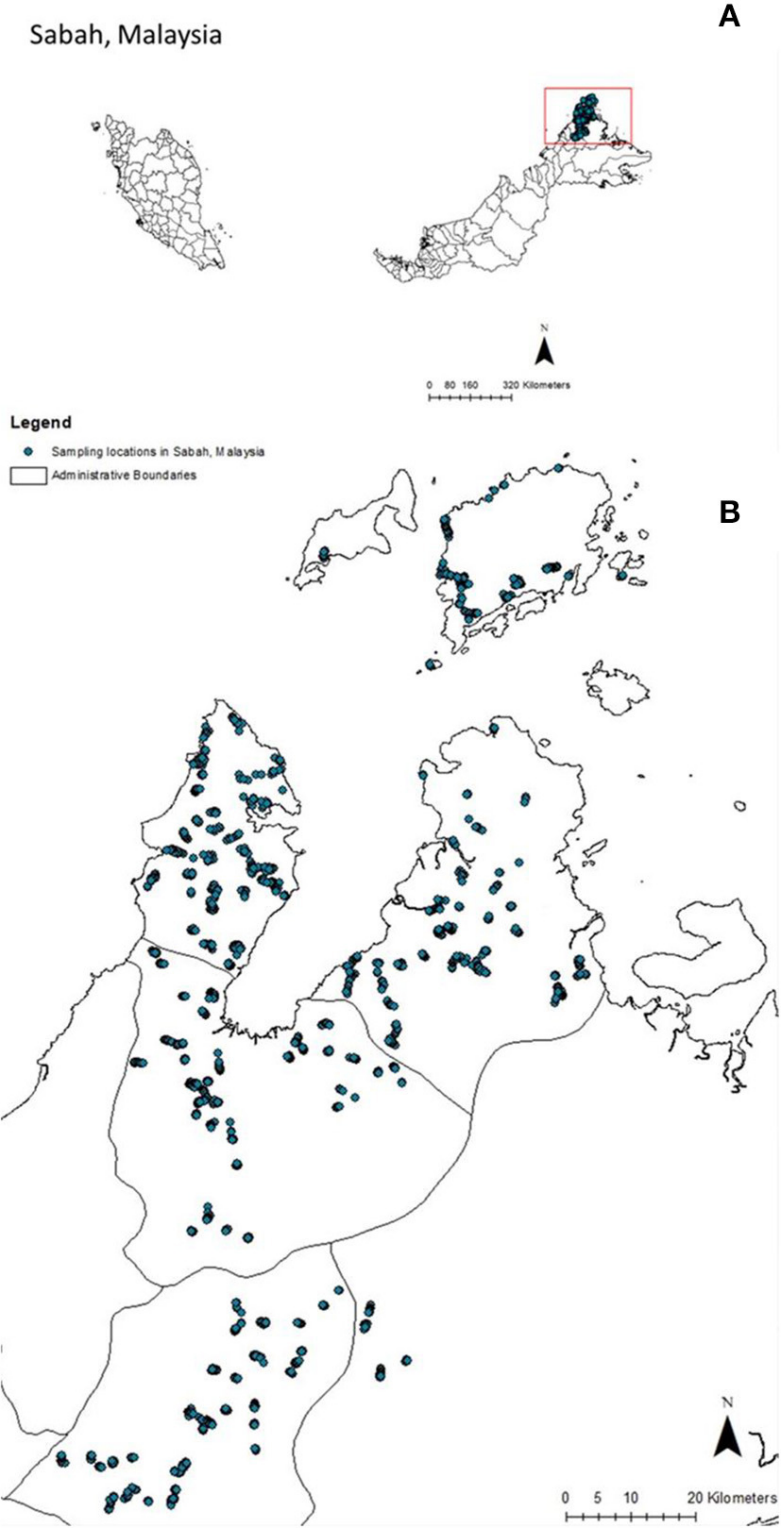
Sampling sites in Sabah, Malaysia. Sampling site locations in Malaysia **(A)** and Sabah state in Malaysia **(B)**.

This study was conducted in four districts of Northern Sabah in Malaysian Borneo (Figure 1). This area is tropical with elevations ranging from sea-level to over 4,000 meters above sea level (MSL). The population is predominantly rural, and most occupations are associated with agricultural or plantation activities. This study was designed to determine the risk factors for malaria using an environmentally stratified, population-based cross-sectional survey that was conducted from September 17, 2015 to December 12, 2015, as described by Fornace et al. (28). Briefly, seroprevalence was estimated using a non-self-weighting two-stage sampling design of 919 villages stratified by forest cover, with a target sample size of 2,650 households and 36 households sampled per village (powered for *Plasmodium knowlesi* seroprevalence). All individuals residing in selected households were asked to participate (ages 3 months−105 years). Finger prick blood sampling was used to prepare blood spots of filter paper (3MM, Whatman, Maidstone, UK).

### Ethics approval

The Medical Research Sub-Committee of the Malaysian Ministry of Health (NMRR-14-713-21117) and the Research Ethics Committee of the London School of Hygiene and Tropical Medicine (8340) approved the Malaysian study and written informed consent was obtained from all study participants. Because CDC authors did not interact with study participants and had no access to personal identifying information, they were determined to be “not engaged” in human subjects research.

### Multiplex IgG detection assay

The IgG responses to 12 antigens from six pathogens were assayed (Table 1; Supplementary Table 1). Merozoite surface protein 1-19 (MSP1-19) and apical membrane antigen-1 (AMA-1) antigens from *Plasmodium falciparum* and *P. vivax* were also included with appropriate control sera as internal positive controls. Excluding the malaria proteins, all antigen-coupled microspheres were provided by the Centers for Disease Control and Prevention (Atlanta, GA, USA) and coupled according to standard Luminex protocols to minimize the signal-to-noise ratio (29). Malaria antigen coupling was optimized in-house as described previously (28, 30) (Luminex Corporation, Austin, TX, USA).

**Table 1:** Cut-off method, seroprevalence and vaccine exposure in percentages, and number of individuals per antigen.

**Antigen, pathogen**	**Disease**	**Percent seroprevalence with 95% CI**	***n***
**Gaussian mixture model (2 distributions)**
Bm33, *Brugia malayi*	Lymphatic Filariasis	10.9 (10.2, 11.6)	8129
Wb123, *Wucheria Bancrofti*	Lymphatic Filariasis	1.7 (1.5, 2.02)	8128
NIE, *Strongyloides stercoralis*	Strongyloidiasis	16.8 (16.7, 16.9)	8131
SAG2A, *Toxoplasma gondii*	Toxoplasmosis	29.9 (28.9, 30.1)	7430
Rp17, *Treponemal pallidum pertenue*	Yaws	4.9 (3.9, 6.1)	1529
TmpA, *Treponemal pallidum pertenue*	Yaws	4.8 (4.00, 5.8)	1660
Rp17 TmpA double positive	Yaws	1.2 (0.7, 1.8)	1638*
VSP3 + VSP5, *Giardia duodenalis*	Giardiasis	23.2 (22.3-24.2)	7682
***K*****-means clustering (*****k*** **=** **3)**
Bm14+BmR1, *Brugia malayi*	Lymphatic Filariasis	3.5 (3.1, 4.00)	6855
Pgp3+Ct694, *Chlamydia trachomatis*	Trachoma	4.5 (3.7, 5.5)	1970

Test samples were eluted from a 3-mm dried blood spot (DBS) punch, corresponding to 2.1 μl of whole blood, and shaken overnight at room temperature in 200 μl of elution buffer (1xPBS, 0.05% sodium azide and 0.05% Tween-20), resulting in a 1:200 pre-dilution, assuming 50% hematocrit. At least 1 day prior to testing, samples were diluted to a final 1:400 dilution using Luminex buffer B (1xPBS, 0.05% Tween, 0.5% BSA, 0.02% sodium azide, 0.1% casein, 0.5% polyvinyl alcohol (PVA), 0.5% polyvinyl pyrrolidone (PVP) and 15.25 μg/ml *E. coli* extract) to prevent non-specific binding. Negative and positive controls were also incubated in buffer B at least 1 day before testing, with negative controls prepared at 1:400, a pooled *P. falciparum* positive prepared at 1:400 and 1:4,000, and a pooled *P. vivax* positive control prepared in a 6-point 2-fold serial dilution (1:400–1:12,800). Fifty microliter of the samples were co-incubated with antigen-coupled beads in a 1-day multiplex serological assay described previously (30). Using a Luminex MAGPIX bioanalyzer and xPONENT software (version 4.2), the background-adjusted median fluorescent intensity (MFI) of wells achieving at least a 30-bead count per analyte were recorded. The *P. vivax* control curve was included on each plate to standardize data between plates (28).

### Determination of seropositivity

Different cut-off approaches were used to determine seropositivity per antigen (Supplementary Table 2). To determine seropositivity, antigen-specific cut-off values from log transformed MFI with background subtracted (MFI-bg) were calculated in R using the mixtools package (31). Gaussian mixture models using the mean of the lower component plus three standard deviations were used to determine cut-off thresholds for eight antigens on this panel. To ensure sufficient negatives for estimating population level exposure, we included individuals of all ages in cut-off determination for LF (Bm33, LF Wb123) and toxoplasma (SAG2A) antigens (32). Data from individuals <3, 5, and 14 years of age were used to determine cut-offs for antigens of strongyloides (NIE), giardia (VSP3, VSP5), and yaws (Rp17, TmpA), respectively. For giardia and yaws, we examined double seropositivity as an indicator of more recent exposure.

As multiple antigens were measured for specific diseases, we also analyzed highly correlated antigens (Pearson's correlation co-efficient > 0.65) for the same pathogen together as representative of individuals exposed to the same pathogen (Supplementary Figure 1). This was done for lymphatic filariasis (Bm14 and BmR1) and trachoma (Pgp3 and Ct694) antigens using K-means clustering (three clusters, highest cluster of MFI responses to multiple antigens are considered seropositive) to classify seropositive and seronegative. We limited analysis of antigens for trachoma to children under 10 years old to exclude sexually acquired venereal chlamydia.

### Statistical analysis of risk factors

We assessed eight demographic, health, and socioeconomic risk factors (Supplementary Table 3). Logistic regression was used to evaluate risk factors association to seroprevalence for each antigen, with household included as a random effect to control for sampling design. Associations with a *p* < 0.05 were considered statistically significant using adjusted odds ratios (Supplementary Table 4). Variables were assessed using variation inflation factor <5 to assess for potential collinearity, and final models were selected using backwards elimination (*p* < 0.05).

### Spatial patterns of exposure risks

To assess the spatial distribution of exposure risks, we additionally assembled potential spatial environmental covariates, including topographic measures, distance to land cover and forest types, population density, accessibility, and climatic variables (Table 2). Pearson correlation analysis was used to exclude highly correlated variables (correlation coefficient > 0.7) with the final dataset including 21 potential spatial and environmental predictors (Supplementary Tables 5, 6). As demographic data was not available for all locations within this region, we did not include additional questionnaire data. All covariates were resampled to 500 m resolution for predictions.

**Table 2:** Study site characteristics.

**Demographic variable**	***n***
Study population	8,205
Males	3,389
Females	4,312
Mean age in years (range)	29 (0–105)
**Occupation**	***n***
Farmer	1,153
Student	3,745
Other occupation	997
No occupation	3,745
**Ethnic groups in Malaysia**	***n***
Bajau	752
Dusun	4,137
Other	1,135
Rungus	2,091
**Environmental variable**	**Mean (range)**
Population density (per km^2^)	1.8 (0–183.4)
Elevation (meters above sea level)	166.4 (4.0–1,258.0)
NDVI	0.5 (-0.2 to 0.9)
Average temperature, 1970–2000 (°C)	26.7 (21.5–27.5)
Mean diurnal range, 1970–2000 (°C)	8.2 (7.00–10.3)
Maximum temperature of warmest month, 1970–2000 (°C)	31.9 (28.1–32.8)
Minimum temperature of coldest month, 1970–2000 (°C)	21.5 (14.8–22.9)
Precipitation of the wettest month. 1970–2000 (mm)	2,417 (2,167–2,754)
Precipitation seasonality, 1970–2000 (coefficient of variation)	44.1 (16.9–59.5)
Distance to intact forest (m)	3,647 (0–19,836)
Distance to irrigated farmland (m)	2,794 (0–23,716)
Distance to oil palm plantation (m)	1098 (0–20,940)

Using the seropositivity thresholds defined above, we fit geostatistical models of household seroprevalence for each disease separately. Models were fit in a Bayesian framework with *p*(*x*_*i*_) denoting the seroprevalence at locations *x*_*i*_, *i* = 1…*n*, with *m*_*i*_ individuals sampled per household location. The full model was specified as:


$$Y_{i}\;\sim \text{ Binomial}\left({\text{m}}_{\text{i}},\text{ p}({\text{x}}_{\text{i}})\right)$$

With the linear predictor for the binomial model specified as:


$$\text{logit}\left(\text{p}({\text{x}}_{\text{i}})\right)\text{= }\beta_{\text{0}}\text{+}\text{d}({\text{x}}_{\text{i}}{)}^{\prime }\beta \text{+ }{\text{w}}_{\text{i}}$$

Where β_0_ represents the intercept, $\text{d}({\text{x}}_{\text{i}}{)}^{\prime }\beta$ represents a vector of location specific covariate effects and *w*_*i*_ represents the spatial effect. Residual spatial autocorrelation was assessed using Moran's I, with spatial effects modeled as a Matern covariance function using the stochastic partial differential equation approach implemented in Integrated Nested Laplace Approximation (R-INLA) (33). Weakly informative priors of Normal (0, 100) were used for intercepts and fixed effect coefficients and penalized complexity priors were used for the spatial effect (34). Final models were assessed using the deviance information criteria (DIC) and root mean squared error. Posterior probabilities were estimated using 1,000 posterior samples. Additionally, to visualize the uncertainty around these predictions, we calculated exceedance probabilities using a 10% seroprevalence threshold (35). These exceedance probabilities represent the probability a location exceeds this threshold; locations with exceedance probabilities around 50% represent areas where there is high uncertainty around this threshold. All analysis was conducted in R statistical software (36), with maps visualized in ArcGIS (ESRI, Redlands, USA).

## Results

### Seroprevalence

Cross-sectional serological survey data was available for 10,100 individuals, with varying number of individuals available for analysis based on sample and antigen availability. Seroprevalence estimates of the whole study site in northern Sabah are shown in Table 1. The seroprevalence of LF antigens were highest in Bm33 (10.9%), then Bm14+ BmR1 (3.5%), and lowest in Wb123 (1.7%). Seroprevalence of *Strongyloides* antigen NIE was 16.8%, for *Toxoplasma* antigen SAG2A was 29.9%, and *Giardia* antigens GVSP3 + GVSP5 was 23.2%. Seroprevalence estimates for yaws antigens in school children <10 years of age for Rp17 was 4.91% and for TmpA was 4.81%. As Rp17 may indicate historical exposure and TmpA may indicate more recent exposure, combined seropositivity to both antigens was 1.2%. Seroprevalence estimates for trachoma Pgp3 +Ct694 were 4.5%.

Study site characteristics that include demographic and environmental variables are listed in Table 2. Seroprevalence of demographic risk factors are listed in Supplementary Table 3. Seropositivity to all assessed antigens showed potential age effects, demonstrating differences in exposure by age category (Supplementary Figures 2–4).

### Risk factor analysis

**Figure 2:**
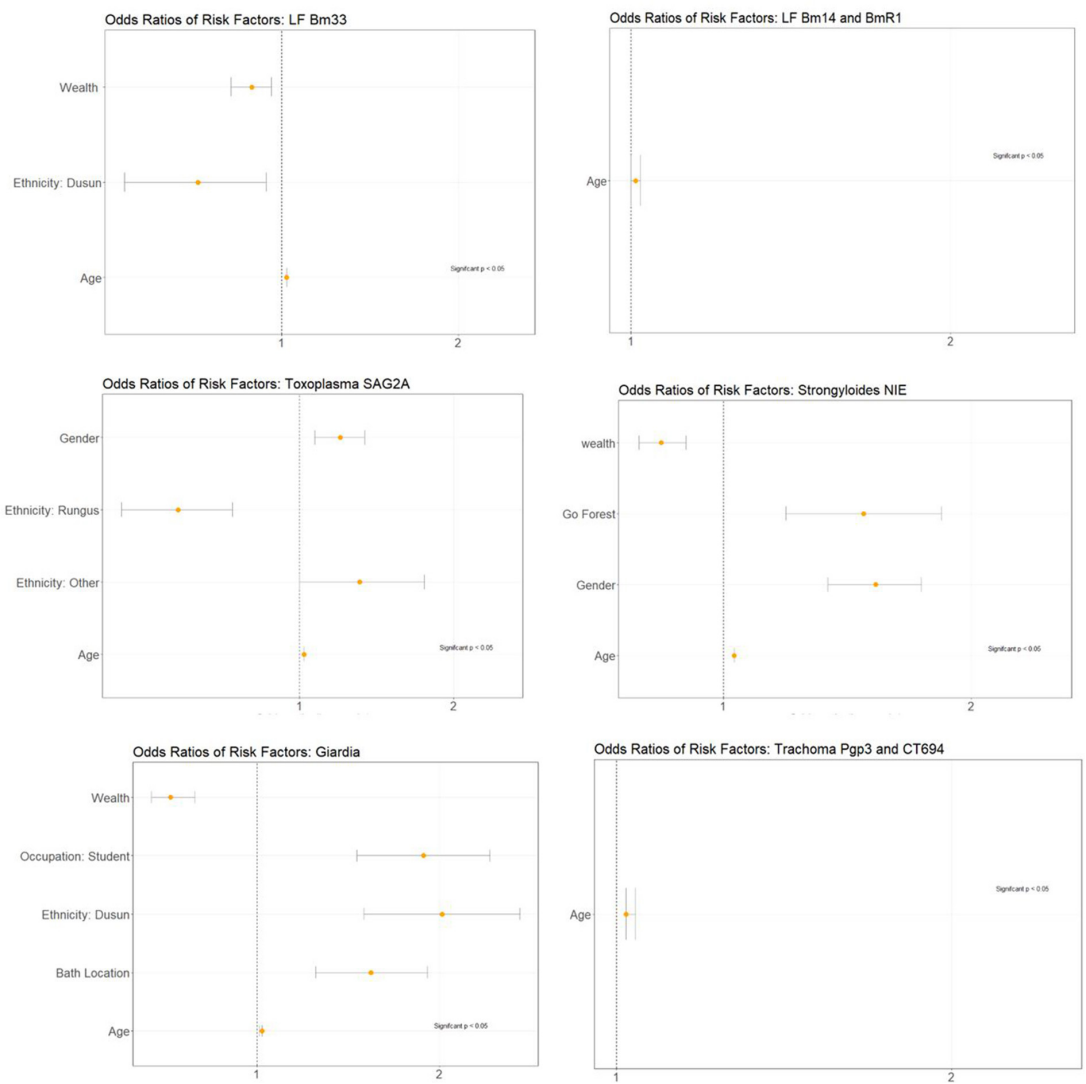
Adjusted odd ratio plots for associated disease risk factors.

Multivariate analysis using logistic regression identified associations between seropositivity and risk factors that were considered significant at *p* < 0.05 (Figure 2; Supplementary Table 4). For LF Bm33 antigen, significant associations were observed for age, wealth, and Dusun ethnicity. Higher socio-economic status and Dusun ethnicity demonstrated decreased odds of risk of exposure. For LF Wb123 antigens, no significant associations were observed, potentially due to the low overall seroprevalence in the population. For LF Bm14 + BmR1, significant associations for risk factors were observed for age only. For toxoplasma SAG2A antigen, significant associations were observed with age, gender, ethnicity, and bath location. Increased odds of exposure were observed for males compared to females and for ethnicity within the “Other” category. Decreased odds of exposure were observed for Rungus ethnic group. For the *Strongyloides* antigen, significant associations were observed for age, wealth, going to the forest, and gender. Higher socio-economic status was associated with decreased odds of exposure, while going to the forest and being male demonstrated increased odds of exposure. For *Giardia* antigens, age, student occupation, Dusun ethnicity, and bath location (i.e., bathing in outdoor locations or with water pipes) were shown to increase odds of exposure, while decreased odds of exposure was observed with higher socio-economic status. For trachoma antigens, age was the only significant risk factor.

### Environmental risk factors and spatial distribution of exposure

**Figure 3:**
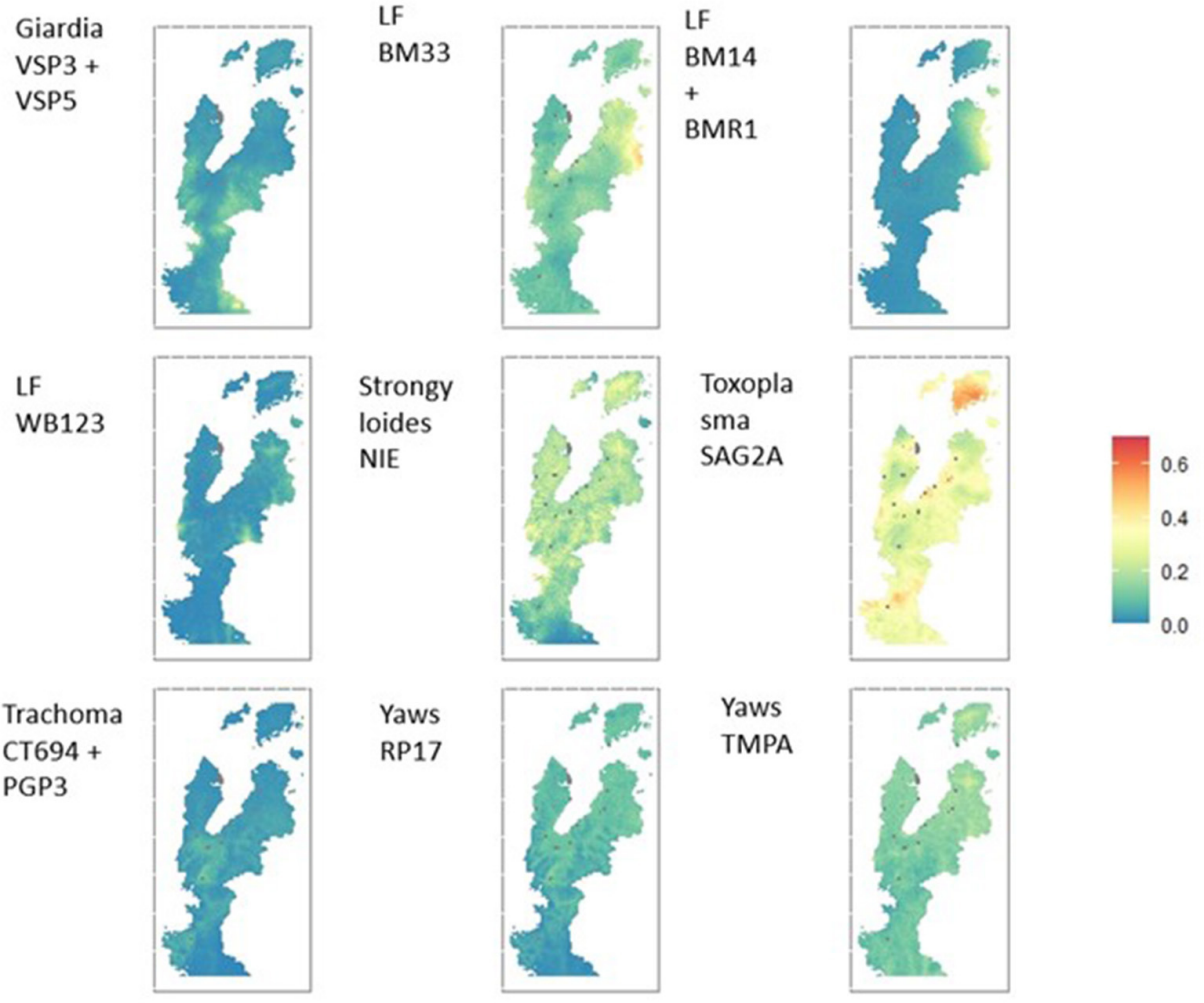
Geostatistical maps showing mean posterior estimated seroprevalence per antigen.

The spatial distribution of seroprevalence of antigens are presented in Figure 3 and Supplementary Figure 6. The study area represented a wide range of ecologies with varying land cover, topography, and population densities (Table 2; Supplementary Table 5). Using these data, we additionally identified predictive spatial and environmental factors for exposure to diseases (Supplementary Table 6). Geostatistical models identified marked differences in the spatial distribution of exposure to the different antigens, revealing areas of potential persistent exposure. Mean posterior estimates of seroprevalence for each pathogen are shown in Figure 3, with estimates of the probability of over 10% of the population being exposed to a particular pathogen shown in Supplementary Figure 5. For example, the spatial distribution of seroprevalence for strongyloides NIE and Yaws TmpA demonstrated broad homogeneity in community exposure, while the spatial distribution of toxoplasma SAG2A and LF BM antigens identified areas of higher seroprevalence compared to the rest of the community.

## Discussion

Serological surveys provide a platform for integrated monitoring of numerous pathogens. In our study, we applied multiplex bead assays to assess seroprevalences and associated risk factors to six NTDs. The seroprevalence results provided evidence of exposure for all NTDs in Malaysia during 2015. Integrating this data within a geostatistical framework enables visualization of spatial dispersal of exposure, detecting priority areas for follow up, surveillance, and targeted public health initiatives.

Analysis of disease specific responses allowed identification of risk factors and spatial distribution of exposure for all diseases, showing broad agreement with other sources of epidemiological data. For example, persistent LF transmission and LF MDA was on-going during the year of the survey [World Health Organization (WHO) Global Health Observatory (GHO), accessed August 19, 2020]. Preventative chemotherapy treatments for strongyloidiasis (prevalence = 16.8%) and other STHs were also administered to parts of the country during the same year of this survey (according to WHO GHO, accessed August 19, 2020), and prevalence estimates were similar to previously reported estimates (31.5%, using ELISA) in Malaysia (Orang Asli) (2). Seroprevalence estimate for toxoplasmosis in this study was 29.9% (CI: 28.9–30.1%) and consistent with similar estimates of previous studies (10, 37). For giardiasis, the seroprevalence estimate was 23.24% (CI: 22.31–24.19%), higher than prior estimates using molecular techniques, which varied between 0.2 and 20% (38, 39). Composite antigen responses for trachoma were detected among 4.52% (CI: 3.68–5.53%) of children 1–9 years of age. This is similar to what was previously observed in areas suspected not to be endemic for trachoma in the Pacific Island nations of the Solomon Islands, Fiji, and Vanuatu (40–42).

For LF, trachoma, yaws, and *Giardia*, multiple antigens were included in determining seropositivity. For LF prevalence, estimates varied using antigens of the same pathogen. This may be due to differing immunogenicity of antigens, antibody kinetics as markers of recent or historical exposure, or possible cross reactivity with antibodies elicited by infection with other pathogens (43, 44). For yaws antigens, seroprevalence was 4.91% (CI: 3.93–6.11%) for Rp17 and 4.81% (CI: 3.98–5.79%) for TmpA. We observed a lack of correlation between the two yaws antigen, which may be due to individual antigen function (Supplementary Figure 6). For example, Cooley et al. have found that Rp17 captures long-lived treponemal antibodies, while TmpA can be potentially used to differentiate exposure based on antibody titer concentrations (45). In our study, we presented double seropositivity for both antigens (1.16%, CI: 0.74–1.80). If further information was available on antibody decay rates, more accurate estimates of infection status and time since infection could be identified. For highly correlated antigens of LF and trachoma, we determined seropositivity by applying K-means clustering approach to classify seroprevalence. This approach to classifying antibody responses may potentially enhance seroprevalence approximations by examining multiple highly correlated antigens within the population, thereby maximizing the use of information from multiple antigens.

We examined several risk factors in this study to demonstrate the utility of multiplex bead assays in supporting integrated disease control efforts. Given the age effect on antibody acquisition, we hypothesized that this association would be present among differing concentrations of the antigen levels and age, within our study population. We found age to be associated with seroprevalence for all antigens, indicating increased likelihood for exposure over time. For giardiasis, however, consistent exposure and chronic infection among children and adults may dampen any age effects on seroprevalence. Differences in antibody concentrations in age may support targeted public health initiatives and further examination of historical exposure patterns among age groups.

Previous studies in Malaysia have found associations between low socioeconomic backgrounds and burden of disease, which is attributable to living standards, working conditions and access to health care (46, 47). We hypothesized that high wealth index would be an acceptable indicator of adequate nutrition, better living conditions, clean water, and easier access to health care, thus reducing seroprevalence in higher socio-economic classes for all NTDs and parasitic disease infections (14, 48, 49). We found associations of higher wealth index and decreased seroprevalence among antigens of LF, *Strongyloides*, and *Giardia*, but no associations were observed for *Toxoplasma* or trachoma.

Common socio-demographic risk factors such as gender, ethnicity, education, occupation, toilet usage, and contact with animals have also been previously studied for LF, toxoplasmosis, and giardiasis in Malaysia (5, 15, 37, 50, 51). We examined these potential risk factors for the diseases represented by our panel of antigens. In this study, significant risk of exposure for occupation was not observed for any disease. Previous studies have found limited data on human seroprevalence in relation to animal exposure for toxoplasmosis in Malaysia, including domestic and livestock animals (52), although Ngui et al. found significant associations with seropositivity for individuals coming in close contact with cats and other pets (10). In our study, we did not find any significant associations with owning animals and increased odds of exposure for any disease marker. We also included bath location in this risk factor analysis, as clean water is important in the prevention of diseases such as STHs and giardiasis, and we found significant associations in decreased prevalence with the use of bathrooms compared to outside bathing for giardiasis in this study. Variation in seroprevalence by ethnic groups may be attributable to cultural norms, occupations, genetics, and geographic dispersion that may warrant more detailed investigation of these differences to aid public health initiatives.

In addition to identifying risk factors, we demonstrate how serological data can be used to characterize the spatial distribution of exposure. Simple visualizations of cluster level mean antibody responses can be used to quickly identify clusters with high responses to multiple pathogens. By integrating serological data into geostatistical models, we identified areas with differential exposure of diseases such as filariasis or focalised transmission such as toxoplasmosis; this data can be used to supplement available infection reports to support elimination campaigns and targeted control. Conversely, we also identify diseases with widespread transmission, such as giardiasis. Characterizing these differences in spatial distribution allows development of appropriate control and surveillance strategies for diseases with vastly different transmission levels. Additionally, this provides further data on the immune status of different populations, with potential implications on susceptibility to disease.

Within this study there are several limitations. Serological standards to determine cut-offs have not been established for most pathogens on this panel, and choice of cut-off method may have impacted the accuracy of seroprevalence approximations. Another limitation within the survey is the lack of individual information about survey respondent's migratory status for coastal Sabah, thus it is unclear whether serological responses represent regional or imported cases. Lastly, we applied a non-conventional method to cluster seropositives using k-means algorithm for correlated antigens of the same pathogens. While trachoma estimates were similar to what was found previously, the discrepancy in seroprevalence estimates among mixture models and k-means clustering for LF antigens implores further exploration of using this method paired with clinically confirmed data or gold standard approaches.

Despite these limitations, this study supports the utility of MBAs for simultaneous disease monitoring of diverse pathogens in low transmission settings. As integrated disease management is being adopted in the WHO NTD Roadmap of 2021 (53), MBAs with serological surveys can provide valuable information regarding population exposure and associated socio-demographic or environmental risk factors impacting transmission of numerous co-endemic pathogens.

## Data availability statement

The datasets presented in this study can be found in online repositories. The names of the repository/repositories and accession number(s) can be found in the article/Supplementary material.

## Ethics statement

The studies involving human participants were reviewed and approved by the Medical Research Sub-Committee of the Malaysian Ministry of Health (NMRR-14-713-21117) and the Research Ethics Committee of the London School of Hygiene and Tropical Medicine (8340). Written informed consent to participate in this study was provided by the participants' legal guardian/next of kin.

## Author contributions

CD, KF, and YC conceptualized research questions and analysis. TW and TC were involved in sample collections and survey design. CP and KT ran multiplex bead assays and provided support to analysis. YC and KF performed the analysis. YC wrote manuscript draft. CD, GS, JP, KF, and PL provided critical reviews and revisions of manuscript draft. All authors contributed to the article and approved the submitted version.

## Funding

We acknowledge the UK Medical Research Council, Natural Environment Research Council, Economic and Social Research Council and Biotechnology and Biosciences Research Council for the funding received for this project through the Environmental and Social Ecology of Human Infectious Diseases Initiative (Grant Number G1100796). KF was supported by a Sir Henry Dale fellowship jointly funded by the Wellcome Trust and Royal Society (Grant Number 221963/Z/20/Z).

## Conflict of interest

The authors declare that the research was conducted in the absence of any commercial or financial relationships that could be construed as a potential conflict of interest.

## Publisher's note

All claims expressed in this article are solely those of the authors and do not necessarily represent those of their affiliated organizations, or those of the publisher, the editors and the reviewers. Any product that may be evaluated in this article, or claim that may be made by its manufacturer, is not guaranteed or endorsed by the publisher.

## Author disclaimer

Use of trade names is for identification only and does not imply endorsement by the Public Health Service or by the U.S. Department of Health and Human Services. The findings and conclusions in this report are those of the authors and do not necessarily represent the official position of the U.S. Centers for Disease Control and Prevention or any other institution.
