# Supplementary material for: Assessing seroprevalence and associated risk factors for multiple infectious diseases in Sabah, Malaysia using serological multiplex bead assays
Source: Front Public Health. 2022 Oct 25;10:924316. doi: 10.3389/fpubh.2022.924316 (PMC9641279; doi:10.3389/fpubh.2022.924316)
Supplement: Supplementary file 1 [file Data_Sheet_1.docx]

**Supplementary Information**

**Supplementary Figure 1. Pearson’s correlation among antigens within the study**

**Supplementary Figure 2. MFI response per age category for Lymphatic Filariasis antigens**

**Supplementary Figure 3. MFI response per age category for Giardia, Toxoplasma and Strongyloides antigens**

**Supplementary Figure 4. MFI response per age category for Yaws and Trachoma antigens**

**Supplementary Figure 5. Maps showing exceedance probability of seroprevalence estimates using an arbitrary 10% threshold per antigen**

**Supplementary Figure 6. Logged MFI antigen responses to Yaws Rp17 and TmpA in children less than 10 years of age**

**Supplementary Table 1. Antigen references and sources**

| Pathogen | Antigen | Abbrev. | Original GenBank accession number | Multiplex References | Antigen source |
| --- | --- | --- | --- | --- | --- |
| *Giardia intestinalis* WB strain (A) | Variant-specific surface protein AS8 | VSP3 | XM_001707314/ HM036222 | (1) | CDC/ Jeff Priest |
| *Giardia intestinalis* GS strain (B) | Variant-specific surface protein 42e | VSP5 | AF354538.1/ HM036223 | (1) | CDC/ Jeff Priest |
| *Toxoplasma gondii* | Surface antigen 2A | SAG2A | M33572 | (2, 3) | CDC/ Jeff Priest |
| *Strongyloides stercoralis* |  | NIE | AAB97359 | (4) | CDC/ Jeff Priest |
| *Chlamydia trachomatis* | Hypothetical, T3SS substrate | CT694 |  | (5) | CDC/ D. Martin |
| *Chlamydia trachomatis* | pCT03 ORF | Pgp3 |  | (5) | CDC/ D. Martin |
| *Brugia malayi* | SXP-1 | Bm14 | M95546 | (6) | CDC/ Jeff Priest |
| *Brugia malayi* | Pepsin inhibitor analog AP-1 | Bm33 | L11001 | (7) | CDC/ Jeff Priest |
| *Brugia malayi* |  | BmR1 |  |  |  |
| *Wucheraria bancrofti* | Larval antigen | Wb123 | HQ438580 | (8) | NIH/ T. Nutman |
| *Treponema pallidum* | Treponemal membrane protein A (Yaws) | TmpA | Com. product | (9) | CDC/ D. Martin |
| *Treponema pallidum* | Yaws | rp17 | Com. product | (9) | CDC/ D. Martin |

**Supplementary Table 2. Cut-off methods and cut-off values per antigen**

| **Mixture Model** | **Cut-off value**  **logged MFI** | **Cut-off value**  **MFI** | **Seroprevalence in overall population (%)** |
| --- | --- | --- | --- |
| **Bm33** |  |  |  |
| All ages | 6.57 | 713.37 | 10.9 |
| **Wb123** |  |  |  |
| All ages | 6.06 | 428.38 | 1.72 |
| **Bm14 (compare K means below)** |  |  |  |
| All ages | 7.11 | 1224.14 | 3.56 |
| **BmR1 (compare K means below)** |  |  |  |
| All ages | 8.69 | 5943.18 | 2.46 |
| **Ss NIE** |  |  |  |
| Less than 3 years | 6.00 | 403.4 | 16.8 |
| **Sag2A** |  |  |  |
| All ages | 5.75 | 314.19 | 29.9 |
| **Rp17** |  |  |  |
| Less than 14 years | 5.91 | 368.71 | 4.91* |
| **TmpA** |  |  |  |
| Less than 14 years | 5.77 | 320.54 | 4.87* |
| **RP17+TmpA** |  |  |  |
| Double seropositive |  |  | 1.16 |
| **VSP3 (compare K-means below)** |  |  |  |
| Less than 5 | 5.15 | 172.43 | 38.37 |
| **VSP5 (compare K-means below)** |  |  |  |
| Less than 5 | 5.34 | 208.51 | 29.21 |
| VSP3 + VSP5 |  |  |  |
| Double seropositive |  |  | 23.24 |
| **K-means** |  |  |  |
| **LF Bm14 + BmR1** |  |  | **Seroprevalence %** |
| All ages |  |  | 3.56 |
| **Pgp3 + Ct694** |  |  |  |
| Less than 10 years |  |  | 4.35* |
| **VSP3 + VSP5** |  |  |  |
| All ages |  |  | 3.90 |

**Supplementary Table 3. Seropositivity by antigens of risk factors**

| **Risk Factors (n)** | **Bm33** | **Wb123** | **BmR1+**  **14** | **SAG2A** | **NIE** | **Rp 17** | **TmpA** | **Pgp+Ct** | **VSP3+5** |
| --- | --- | --- | --- | --- | --- | --- | --- | --- | --- |
| **N (8205)** | 8129 | 8128 | 6855 | 7430 | 8131 | 1529 | 1660 | 1970 | 7682 |
| **Gender** |  |  |  |  |  |  |  |  |  |
| **M (3893)** | 11.62  (10.90- 12.29) | 1.87  (1.60-2.19) | 3.96 (3.54-4.45) | 31.26 (30.24-32.35) | 19.69 (18.84-20.56) | 14.31 (12.6-16.0) | 27.20 (25.0-29.4) | 3.32 (2.45-4.14) | 23.67 (22.74-24.65) |
| **F (4312)** | 10.20  (9.54-10.85) | 1.52  (1.33-1.87) | 3.15 (2.69-3.50) | 28.62 (27.57-29.63) | 14.22 (13.44-14.95) | 13.14 (11.4-14.8) | 23.98 (21.9-26.1) | 4.55 (3.52-5.48) | 22.85 (21.86-23.73) |
| **Go Forest** |  |  |  |  |  |  |  |  |  |
| **Y (615)** | 14.43 (13.63-15.17) | 1.97 (1.41-1.98) | 6.11 (5.54-6.65) | 35.50 (36.59-34.41) | 30.00 (29.00-30.99) | 13.90 (12.3-15.7) | 22.12 (20.1-24.1) | 0.00 (0.00-0.00) | 27.58 (26.59-28.60) |
| **N (7538)** | 10.62 (9.93-11.27) | 1.71 (1.69-2.31) | 3.35 (2.98-3.82) | 29.45 (30.44-28.36) | 15.80 (16.59-15.00) | 14.18 (11.9-15.3) | 25.76 (23.7-27.9) | 3.92 (2.97-4.82) | 22.94 (21.95-23.84) |
| **Wealth** |  |  |  |  |  |  |  |  |  |
| **1 (1797)** | 13.00 (12.27 -13.73) | 1.92  (1.60-2.19) | 4.49 (4.02-4.98) | 31.61 (30.54-32.65) | 20.72 (19.82-21.58) | 17.11 (15.2-18.9) | 30.99 (28.8-33.3) | 3.40 (2.54-4.26) | 30.82 (29.76-31.83) |
| **2 (2037)** | 11.34 (10.61-20.12) | 1.83 (1.51-2.09) | 3.95 (3.44-4.35) | 27.38 (26.38-28.41) | 18.13 (17.26-18.94) | 16.83 (14.9-18.7) | 24.59 (22.5-26.7) | 3.82 (2.89-4.71) | 26.28 (25.31-27.28) |
| **3 (2212)** | 10.23 (17.2- 18.91) | 1.92 (1.61-2.68) | 3.56 (3.14-4.05) | 29.23 (28.18-30.21) | 16.57 (15.76-17.43) | 12.95 (11.1-14.9) | 26.01 (23.9-28.1) | 4.69 (3.79-5.61) | 20.40 (19.41-21.38) |
| **4 (2159)** | 9.33  (14.4-16.23) | 1.26 (1.01-1.58) | 2.29 (1.85-2.75) | 31.44 (30.39-32.41) | 12.59 (11.76-13.43) | 8.50 (6.63-10.4) | 21.54 (19.4-23.6) | 3.71  (2.79-4.61) | 17.09 (16.11-18.08) |
| **Occupation** |  |  |  |  |  |  |  |  |  |
| **Farmer (1153)** | 15.91 (15.11-16.69) | 2.20 (1.87-2.52) | 5.67 (5.15-6.24) | 37.12 (35.99-38.20) | 28.21 (27.22-29.18) | - | - | - | 26.46 (25.51-27.49) |
| **None (3745)** | 10.91 (10.22-11.58) | 1.77 (1.51-2.09) | 3.20 (2.78-3.61) | 29.67 (28.66-30.74) | 16.07 (15.29-16.09) | - | - | - | 21.46 (20.57-22.42) |
| **Other (997)** | 12.74 (12.02-13.38) | 1.31 (1.01-1.59) | 4.60 (4.18-5.01) | 35.78 (34.75-36.84) | 23.84 (22.99-24.60) | - | - | - | 21.99 (21.07-22.92) |
| **Student (2245)** | 7.53  (6.82-  8.17) | 1.62 (1.31-1.89) | 2.60 (2.18-3.01) | 23.94 (22.86-24.94) | 9.47 (8.69-10.30) | - | - | - | 25.47 (24.57-26.42) |
| **Ethnicity** |  |  |  |  |  |  |  |  |  |
| **Bajau (752)** | 12.82 (12.08-13.53) | 1.20 (0.96-1.44) | 5.30 (4.77-5.83) | 31.75 (30.74-32.86) | 13.89 (13.41-14.65) | 17.52 (15.6-19.4) | 24.65 (22.5-26.7) | 0.78 (0.37-1.22) | 16.92 (16.05-17.74) |
| **Dusun (4137)** | 9.59 (8.96-10.24) | 1.20 (0.96-1.49) | 2.28 (1.94-2.65) | 30.81 (29.74-31.85) | 17.14 (16.28-17.92) | 11.16 (9.6-12.8) | 22.20 (20.2-24.2) | 4.82 (3.78-5.82) | 26.49 (25.50-27.49) |
| **Other (1135)** | 13.09 (12.45-13.74) | 2.48 (2.26-2.74) | 4.30 (3.95-4.65) | 37.49 (36.44-38.56) | 13.70 (12.88-14.52) | 13.66 (12.1-15.3) | 34.93 (32.9-36.9) | 2.07 (1.07-3.12) | 16.88 (15.90-17.89) |
| **Rungus (2091)** | 11.69 (11.06-12.34) | 2.57 (2.36-2.84) | 4.79 (4.45-5.15) | 23.67 (22.64-24.75) | 19.20 (18.38-20.02) | 16.37 (14.8-17.9) | 26.46 (24.5-28.5) | 3.51 (2.47-4.52) | 22.84 (21.80-23.79) |
| **Bednet** |  |  |  |  |  |  |  |  |  |
| **Y (3175)** | 11.89  (11.19-12.06) | 2.09 (1.78-2.41) | 4.07 (3.63-4.56) | 29.30  (28.26-31.6) | 17.18 (16.38-18.02) | 14.64 (12.4-15.9) | 26.50 (24.6-28.9) | 2.95 (2.19-3.18) | 25.72 (24.71-26.68) |
| **N (4964)** | 10.23 (9.53-10.86) | 1.49 (1.23-2.41) | 3.19 (2.78-3.61) | 30.30 (29.25-31.34) | 16.55 (15.78-17.41) | 13.79 (11.5-14.9) | 24.73 (22.3-26.5) | 4.62 (3.60-5.59) | 21.57 (20.67-22.52 |
| **Animals** |  |  |  |  |  |  |  |  |  |
| **None (1900)** | 10.56 (9.93-11.27) | 1.54 (1.23- 1.76) | 2.64 (2.23-2.97) | 32.21  (31.13-33.26) | 14.38 (13.63-15.16) |  |  | 3.89 (2.98-4.81) | 23.94 (22.94-24.85) |
| **Domestic (1987)** | 10.41 (9.73-11.06) | 1.79 (1.51-2.09) | 3.40 (3.07-3.92) | 27.93 (26.88-38.91) | 17.49 (16.67-18.32) | 28.57  (26.3-30.9) | 38.1 (35.7-40.5) | 6.28 (5.15-7.45) | 23.30 (22.35-24.24) |
| **Farm (4318)** | 11.23 (10.51-18.89) | 1.77 (1.51-2.09) | 3.95 (3.54-4.46) | 29.67 (28.66-30.73) | 17.57 (16.77-25.1) | 13.3 (11.6-15.0) | 25.2 (23.1-27.3) | 2.83 (2.02-3.58) | 22.90 (21.96-23.83) |
| **Bath Location** |  |  |  |  |  |  |  |  |  |
| **Bathroom (5152)** | 10.57 (9.93-11.27) | 1.60 (1.32-1.87) | 3.50 (3.07-3.92) | 30.25 (29.15-31.24) | 15.34 (14.51-16.09) | 12.50 (6.85-18.1) | 25.68 (18.3-33.1) | 4.44 (3.42-5.38) | 20.27 (19.39-21.20) |
| **Outdoors (1638)** | 11.85 (11.19-12.61) | 2.37 (2.07-2.73) | 4.11 (3.63-4.56) | 30.32 (29.25-31.34) | 20.39 (19.52-21.28) | 17.52 (11.0-23.9) | 29.45 (21.8-37.2) | 2.51 (1.75-3.24) | 29.53 (28.47-30.52) |
| **Water Pipe (1381)** | 11.15 (10.49-11.91) | 1.49 (1.16-1.83) | 3.03 (2.53-3.46) | 28.11 (27.05-29.15) | 18.60 (17.72-19.47) | 15.84 (9.32-22.3) | 23.97 (16.3-31.7) | 3.57 (2.86-4.34) | 27.37 (26.37-28.42) |

**Supplementary Table 4. Odds ratios of risk factors for antigens.** P-values of p<0.05 are bolded.

1. Bm33

| **Risk Factors** | **Bm33** | **P Value** |  |  |
| --- | --- | --- | --- | --- |
|  | **Crude OR** |  | **Adjusted OR** | **P-value** |
| **Age** |  |  |  |  |
|  | 1.02(1.02-1.02) | <2e-16 | 1.02 (1.02-1.02) | 2.2e-16 |
| **Gender (F)** |  |  |  |  |
| M | 1.75 (1.01-1.36) | 0.0318 |  |  |
| **Go Forest (N)** |  |  |  |  |
| Y | 1.48 (1.14-1.92) | 0.00319 |  |  |
| **Wealth (1)** |  |  |  |  |
| 2 | 0.85 (0.68-1.08) |  |  |  |
| 3 | 0.73 (0.57-0.92) | 0.0071 |  |  |
| 4 | 0.66 (0.52-0.84) | 0.0006 | 0.89 (0.82-0.96) | 0.002076 |
| **Occupation (Farmer)** |  |  |  |  |
| None | 0.15 (0.49-0.75) | 3.56e-06 |  |  |
| Other | 0.72 (0.55-0.94) | 0.0169 |  |  |
| Student | 0.40 (0.31-0.51) | 8.54e-14 |  |  |
| **Ethnicity (Bajau)** |  |  |  |  |
| Dusun | 0.73 (0.55 -0.97) | 0.0306 | 0.72 (0.54-0.94) | 8.72e-05 |
| Other | 1.05 (0.75-1.45) |  | 1.01 (0.74-1.39) |  |
| Rungus | 0.92 (0.68-1.24) |  | 0.85 (0.63-1.14) |  |
| **Animals (N)** |  |  |  |  |
| Domestic | 0.97 (0.76-1.24) |  |  |  |
| Farm | 1.07 (0.87-1.31) |  |  |  |
| **Bath Location (Bathroom)** |  |  |  |  |
| Outdoor | 1.15 (0.94- 1.42) |  |  |  |
| Water pip | 1.11 (0.88 -1.39) |  |  |  |
| **Bednet (N)** |  |  |  |  |
| Y | 1.21 (1.02- 1.42) | 0.0253 |  |  |

1. Wb123

| **Risk Factors** | **Wb123** |  |  |  |
| --- | --- | --- | --- | --- |
|  | **Crude OR** | **P Value** | **Adjusted OR** | **P-value** |
| **Age** |  |  |  |  |
|  | 1.02 (1.01-1.02) | <2e-16 |  |  |
| **Gender (F)** |  |  |  |  |
| M | 1.05(1.04-1.06) | <2e-16 |  |  |
| **Go Forest (N)** |  |  |  |  |
| Y | 1.08 (0.44-2.65) |  |  |  |
| **Wealth (1)** |  |  |  |  |
| 2 | 0.94 (0.42-2.09) |  |  |  |
| 3 | 0.98 (0.97-0.98) | <2e-16 |  |  |
| 4 | 0.68 (0.68-0.68) | <2e-16 |  |  |
| **Occupation (Farmer)** |  |  |  |  |
| None | 0.70 (0.36-1.36) |  |  |  |
| Other | 0.39 (0.14-1.07) |  |  |  |
| Student | 0.45 (0.22-0.92) | 0.0304 |  |  |
| **Ethnicity (Bajau)** |  |  |  |  |
| Dusun | 1.35 (0.34-5.96) |  |  |  |
| Other | 4.52 (1.01-20.17) | 0.0484 |  |  |
| Rungus | 2.73(0.62-11.95) |  |  |  |
| **Animals (N)** |  |  |  |  |
| Domestic | 1.09 (1.08-1.01) | <2e-16 |  |  |
| Farm | 1.09(0.62-1.92) |  |  |  |
| **Bath Location (Bathroom)** |  |  |  |  |
| Outdoor | 1.48 (0.67-3.61) |  |  |  |
| Water pip | 1.71 (0.69-4.24) |  |  |  |
| **Bednet (N)** |  |  |  |  |
| Y | 0.99 (0.56-1.77) |  |  |  |

1. BmR1 and Bm14

| **Risk Factors** | **BmR1 and Bm14** |  |  |  |
| --- | --- | --- | --- | --- |
|  | **Crude OR** |  | **Adjusted OR** |  |
| **Age** |  |  |  |  |
|  | 1.03 (1.02-1.03) | 1.71e-08 | 1.01 (1.01-1.02) | 5.65e-08 |
| **Gender (F)** |  |  |  |  |
| M | 2.99 (1.64-5.48) |  |  |  |
| **Go Forest (N)** |  |  |  |  |
| Y | 2.99 (1.64-5.47) | 0.000353 |  |  |
| **Wealth (1)** |  |  |  |  |
| 2 | 0.87(0.39-1.95) |  |  |  |
| 3 | 0.68 (0.29- 1.56) |  |  |  |
| 4 | 0.465(0.18- 1.12) |  |  |  |
| **Occupation (Farmer)** |  |  |  |  |
| None | 0.36 (0.21-0.60) | 8.76e-05 |  |  |
| Other | 0.65 (0.34-1.26) |  |  |  |
| Student | 0.28 (0.16-0.50) | 1.60e-05 |  |  |
| **Ethnicity (Bajau)** |  |  |  |  |
| Dusun | 0.45 (0.17-1.18) |  | 0.37 (0.19-0.71) | 1.135e-06 |
| Other | 0.83 (0.30-2.37) |  | 0.92 (0.48-1.78) |  |
| Rungus | 0.69 (0.26-1.81) |  | 0.72 (0.38 -1.38) |  |
| **Animals (N)** |  |  |  |  |
| Domestic | 1.39 (0.54-3.57) |  |  |  |
| Farms | 1.53 (0.68-3.46) |  |  |  |
| **Bath Location (Bathroom)** |  |  |  |  |
| Outdoor | 1.15 (0.58-2.28) |  |  |  |
| Water pip | 0.78 (0.36—1.70) |  |  |  |
| **Bednet (N)** |  |  |  |  |
| Y | 0.96 (0.56-1.66) |  |  |  |

1. SAG2A

| **Risk Factors** | **SAG2A** |  |  |  |
| --- | --- | --- | --- | --- |
|  | **Crude OR** |  | **Adjusted OR** |  |
| **Age** |  |  |  |  |
|  | 1.02 (1.02-1.02) | <2e-16 | 1.02 (1.02-1.02) | 2.2e-16 |
| **Gender (F)** |  |  |  |  |
| M | 1.15 (1.15-1.16) | <2e-16 | 1.20 (1.07-1.34) | 0.00177 |
| **Go Forest (N)** |  |  |  |  |
| Y | 1.39 (1.37-1.38) | <2e-16 |  |  |
| **Wealth (1)** |  |  |  |  |
| 2 | 0.79 (0.66-0.79) | 0.0132 |  |  |
| 3 | 0.88 (0.73-1.05) |  |  |  |
| 4 | 0.98 (0.82-1.18) |  |  |  |
| **Occupation (Farmer)** |  |  |  |  |
| None | 0.67 (0.67-0.67) | <2e-16 |  |  |
| Other | 0.93 (0.93-0.93) | <2e-16 |  |  |
| Student | 0.50 (0.50-0.50) | <2e-16 |  |  |
| **Ethnicity (Bajau)** |  |  |  |  |
| Dusun | 0.92 (0.74-1.16) |  | 0.89 (0.71-1.13) | 4.71e-13 |
| Other | 1.32 (1.02-1.72) | 0.036488 | 1.31 (1.00-1.75) |  |
| Rungus | 0.62 (0.48-0.79) | 0.000112 | 0.58 (0.45-0.74) |  |
| **Animals (N)** |  |  |  |  |
| Domestic | 0.79(0.79-0.79) | <2e-16 |  |  |
| Farm | 0.88 (0.88-0.88) | <2e-16 |  |  |
| **Water Bath Location (Bathroom)** |  |  |  |  |
| Outdoor | 1.02 (0.86-1.20) |  |  |  |
| Water pip | 0.90 (0.76-1.07) |  |  |  |
| **Bednet (N)** |  |  |  |  |
| Y | 0.98 (0.97-0.98) |  |  |  |

1. NIE

| **Risk Factors** | **NIE** |  |  |  |
| --- | --- | --- | --- | --- |
|  | **Crude OR** |  | **Adjusted OR** | **P-value** |
| **Age** |  |  |  |  |
|  | 1.03 (1.03-1.04) | <2e-16 | 1.03 (1.03-1.05) | < 2e-16 |
| Gender (F) |  |  |  |  |
| M | 1.51 (1.33-1.71) | 7.29e-11 | 1.53 (1.34-1.74) | 3.14e-10 |
| Go Forest (N) |  |  |  |  |
| Y | 2.44 (1.98-3.01) | <2e-16 | 1.48 (1.19-1.84) | 0.000442 |
| Wealth (1) |  |  |  |  |
| 2 | 0.22 (0.19-0.26) |  | 0.84 (0.79-0.90) | 2.53e-07 |
| 3 | 0.76 (0.63-0.92) | 0.00425 |  |  |
| 4 | 0.53 (0.43-0.65) | 8.61e-10 |  |  |
| Occupation (Farmer) |  |  |  |  |
| None | 0.45 (0.38-0.54) | <2e-16 |  |  |
| Other | 0.78 (0.63-0.97) | 0.0269 |  |  |
| Student | 0.24 (0.19-0.30) | <2e-16 |  |  |
| Ethnicity (Bajau) |  |  |  |  |
| Dusun | 1.27 (0.98-1.64) |  |  |  |
| Other | 0.96 (0.71-1.30) |  |  |  |
| Rungus | 1.49 (1.13-1.94) | 0.00407 |  |  |
| Animals (N) |  |  |  |  |
| Domestic | 1.28 (1.05-1.58) | 0.01698 |  |  |
| Farm | 1.30 (1.09-1.56) | 0.00365 |  |  |
| Bath Location (Bathroom) |  |  |  |  |
| Outdoor | 1.44 (1.22-1.72) | 1.59e-05 |  |  |
| Water pip | 1.26 (1.05 -1.52) | 0.0112 |  |  |
| Bednet (N) |  |  |  |  |
| Y | 1.05 (0.91-1.21) |  |  |  |

1. VSP3 and VSP5

| **Risk Factors** | **VSP3 and VSP5** |  |  |  |
| --- | --- | --- | --- | --- |
|  | **Crude OR** |  | **Adjusted OR** | **P-value** |
| **Age** |  |  |  |  |
|  | 1.02 (1.01-1.02) | < 2e-16 | 1.02 (1.01-1.02) | < 2e-16 |
| **Gender (F)** |  |  |  |  |
| M | 1.08 (0.95-1.22) |  |  |  |
| **Go Forest (N)** |  |  |  |  |
| Y | 1.31 (1.03-1.66) |  |  |  |
| **Wealth (1)** |  |  |  |  |
| 2 | 0.78 (0.63-0.97) | 0.0258 | 0.72 (0.67-0.79) | 5.07e-13 |
| 3 | 0.51 (0.41-0.63) | 2.94e-09 |  |  |
| 4 | 0.39 (0.31-0.49) | 9.53e-16 |  |  |
| **Occupation (Farmer)** |  |  |  |  |
| None | 0.72 (0.59-0.87) | 0.000815 | 0.97 (0.78-1.18) |  |
| Other | 0.83 (0.64-1.06) |  | 1.09 (0.85-1.43) |  |
| Student | 0.96 (0.78-1.18) |  | 1.88 (1.46-2.42) | 9.32e-07 |
| **Ethnicity (Bajau)** |  |  |  |  |
| Dusun | 1.76 (1.33-2.34) | 8.33e-05 | 2.02 (1.50-2.71) | 3.94e-06 |
| Other | 0.97 (0.69-1.36) |  | 0.97 (0.69- 1.37) |  |
| Rungus | 1.47 (1.09-1.99) | 0.0119 | 1.39 ( 1.02- 1.91) |  |
| **Animals (N)** |  |  |  |  |
| Domestic | 0.91 (0.72-1.15) | 0.0400 | 0.76 (0.60-0.98) | 0.7511 |
| Farm | 0.96 (0.79-1.18) | 0.040 | 0.74 (0.61-0.92) |  |
| **Bath Location (Bathroom)** |  |  |  |  |
| Outdoor | 1.87 (1.55-2.26) | 7.33e-11 | 1.54 (1.25-1.91) | 4.70e-05 |
| Water pip | 1.49 (1.21- 1.83) | 0.000122 | 1.09 (0.87-1.36) |  |
| **Bednet (N)** |  |  |  |  |
| Y | 1.32 (1.13-1.54) | 0.000329 |  |  |

1. Rp17

| **Risk Factors** | **Rp17** |  |  |
| --- | --- | --- | --- |
|  | Crude OR | **Adjusted OR** |  |
| **Age** |  |  |  |
|  | 1.03 (1.02-1.04) |  |  |
| **Gender (F)** |  |  |  |
| M | 0.65 (0.46-0.92) |  |  |
| **Go Forest (N)** |  |  |  |
| Y | 0.00 (0.50-2.53) |  |  |
| **Wealth (1)** |  |  |  |
| 2 | 1.02 (0.58-1.79) |  |  |
| 3 | 1.48 (0.84-2.59) |  |  |
| 4 | 1.13 (0.63-1.99) |  |  |
| **Occupation (Farmer)** |  |  |  |
| None | - | - |  |
| Other | - | - |  |
| Student | - | - |  |
| **Ethnicity (Bajau)** |  |  |  |
| Dusun | 0.49 (0.24-0.96) | 0.44 (0.22-0.89) | 5.726e-06 |
| Other | 0.64 (0.29-1.41) | 0.66 (0.31-1.42) |  |
| Rungus | 0.63 (0.31-1.27) | 0.59 (0.28-1.25) |  |
| **Animals (N)** |  |  |  |
| Y | - | - |  |
| **Bath Location (Bathroom)** |  |  |  |
| Outdoor | 0.86 (0.43-1.75) |  |  |
| Water pip | 0.89 (0.51-1.53) |  |  |
| **Bednet (N)** |  |  |  |
| Y | 0.71 (0.47-1.05) |  |  |

1. Ct694 and Pgp3

| **Risk Factors** | **Pgp3 and Ct694** |  |  |  |
| --- | --- | --- | --- | --- |
|  | **Crude OR** |  | **Adjusted OR** | **P-value** |
| **Age** |  |  |  |  |
|  | 1.07 (0.88-1.31) | 0.494 | 1.02 (1.02-1.04) | 1.26e-13 |
| **Gender (F)** |  |  |  |  |
| M | 0.42 (0.15-0.12) |  |  |  |
| **Go Forest (N)** |  |  |  |  |
| Y | - |  |  |  |
| **Wealth (1)** |  |  |  |  |
| 2 | 1.02 (0.08- 12.09) |  |  |  |
| 3 | 1.01 (0.09-13.50) |  |  |  |
| 4 | 0.96 (0.07-12.38) |  |  |  |
| **Occupation (Farmer)** |  |  |  |  |
| None | - |  |  |  |
| Other | - |  |  |  |
| Student | - |  |  |  |
| **Ethnicity (Bajau)** |  |  |  |  |
| Dusun | 1.02 (0.08-12.09) |  |  |  |
| Other | 1.10 (0.09-13.31) |  |  |  |
| Rungus | 0.96 (0.08-12.37) |  |  |  |
| **Animals (N)** |  |  |  |  |
| Domestic | - |  |  |  |
| Farm | - | |  |  |
| **Bath Location (Bathroom)** |  |  |  |  |
| Other | 0.17 (0.00-5.03) |  |  |  |
| Water pip | 0.14 (0.00-1.60) |  |  |  |
| **Bednet (N)** |  |  |  |  |
| Y | 1.25 (0.27-5.78) |  |  |  |

**Supplementary Table 5. Spatial and environmental covariates**

| Parameter | Description | Resolution | Source |
| --- | --- | --- | --- |
| Elevation | Elevation (metres above sea level) | 30 m | ASTER Global Digital Elevation Map (10) |
| Slope and aspect | Slope and aspect (degrees) | 30 m | Calculated from ASTER Global Digital Elevation Map |
| TWI | Topographic wetness index | 30m | Calculated from ASTER Global Digital Elevation Map |
| NDVI | Normalised differential vegetation index | 30 m | Calculated from NIR and Red Landsat8 bands (11) |
| Land cover | Euclidean distance to different land cover types | 30 m | Calculated from land cover map of study site, described by (12) |
| Location of roads and houses | GPS coordinates | 5 m | Mapped during GPS field surveys, described by (12) |
| Distance to roads and houses | Distance from nearest road and house | 30 m | Calculated from GPS coordinates |
| Population density | UN-adjusted 2015 population density | 100 m | World Pop (13) |
| Bioclimatic variables | Bioclimatic indicators of ecology, 1970 - 2000 | 1000 m | Calculated from (14) |

**Supplementary Table 6. Mean posterior estimates of coefficients of fixed effects and spatial range for geostatistical models for household seroprevalence to:**

**a. LF WB123**

| Covariate* | Mean | 95% Bayesian Credible Interval (BCI) | |
| --- | --- | --- | --- |
|  |  | 2.5% | 97.5% |
| NDVI | 0.138 | -0.021 | 0.300 |
| Mean diurnal range | -2.713 | -5.096 | -0.143 |
| Maximum temperature | 1.626 | -0.299 | 3.496 |
| Minimum temperature | -2.514 | -5.883 | 0.984 |
| Precipitation | 0.371 | -0.359 | 0.876 |
| Seasonality | -1.403 | -2.807 | -0.116 |
| Distance to bush forest | 0.382 | 0.055 | 0.689 |
| Distance to old forest | 0.128 | -0.148 | 0.371 |
| Spatial range (km) | 86.17 | 75.31 | 96.64 |

* All covariates mean-centered and squared

**b. LF BM14/ BMR1**

| Covariate* | Mean | 95% BCI | |
| --- | --- | --- | --- |
|  |  | 2.5% | 97.5% |
| Elevation | 1.048 | -0.517 | 2.612 |
| Maximum temperature | 0.734 | -0.445 | 1.911 |
| Distance to mangroves | -1.039 | -2.576 | 0.496 |
| Population density ^ 2 | -0.199 | -0.444 | 0.047 |
| Spatial range (km) | 183.9 | 40.63 | 592.73 |

* All covariates mean-centered and squared

**c. LF BM33**

| Covariate* | Mean | 95% BCI | |
| --- | --- | --- | --- |
|  |  | 2.5% | 97.5% |
| Average temperature | -0.799 | -3.103 | 1.281 |
| Mean diurnal range | 0.627 | -0.905 | 2.291 |
| Population density ^ 2 | -0.218 | -0.495 | 0.002 |
| Spatial range (km) | 197.31 | 42.435 | 630.12 |

* All covariates mean-centered and squared

**d. Giardia**

| Covariate* | Mean | 95% BCI | |
| --- | --- | --- | --- |
|  |  | 2.5% | 97.5% |
| NDVI | -0.064 | -0.179 | 0.050 |
| Distance from sea | 2.082 | 0.453 | 3.709 |
| Average temperature | -3.854 | -6.950 | -0.761 |
| Mean diurnal range | -1.905 | -3.599 | -0.213 |
| Maximum temperature | 2.368 | 0.153 | 4.582 |
| Distance from agricultural land | -1.820 | -3.017 | -0.625 |
| Spatial range (km) | 21.76 | 4.810 | 64.255 |

* All covariates mean-centered and squared

**e. Strongyloides**

| Covariate* | Mean | 95% BCI | |
| --- | --- | --- | --- |
|  |  | 2.5% | 97.5% |
| NDVI | 0.060 | 0.007 | 0.112 |
| Elevation | -0.879 | -1.659 | -0.099 |
| Average temperature | -0.827 | -1.626 | -0.028 |
| Mean diurnal range | -0.118 | -0.299 | 0.063 |
| Spatial range (km) | 3.54 | 1.604 | 6.365 |

* All covariates mean-centered and squared

**f. Trachoma (under 10 years old)**

| Covariate* | Mean | 95% BCI | |
| --- | --- | --- | --- |
|  |  | 2.5% | 97.5% |
| Aspect | -0.091 | -0.200 | 0.014 |
| Elevation | 1.349 | -0.511 | 3.221 |
| Distance from sea | -2.380 | -4.134 | -0.652 |
| Mean diurnal range | 3.422 | 1.460 | 5.398 |
| Minimum temperature | 2.672 | 0.121 | 5.281 |

* All covariates mean-centered and squared

**g. Yaws RP17 (under 10 years old)**

| Covariate* | Mean | 95% BCI | |
| --- | --- | --- | --- |
|  |  | 2.5% | 97.5% |
| Slope | -0.110 | -0.257 | 0.038 |
| Distance from the sea | -0.973 | -1.615 | -0.332 |
| Maximum temperature | 1.023 | 0.414 | 1.632 |
| Minimum temperature | -1.242 | -2.165 | -0.319 |
| Spatial range (km) | 6.541 | 0.320 | 31.223 |

* All covariates mean-centered and squared

**h. Yaws TMPA (under 10 years old)**

| Covariate* | Mean | 95% BCI | |
| --- | --- | --- | --- |
|  |  | 2.5% | 97.5% |
| Mean diurnal range | -1.181 | -2.118 | -0.256 |
| Maximum temperature | 0.749 | -0.009 | 1.513 |
| Minimum temperature | -1.142 | -2.528 | 0.231 |
| Precipitation | 0.094 | 0.014 | 0.173 |
| Distance from sparse forest | 0.149 | 0.021 | 0.274 |
| Distance from mangroves | 0.451 | -0.027 | 0.933 |
| Distance from irrigated crops | -0.144 | -0.324 | 0.028 |

* All covariates mean-centered and squared

**i. Toxoplasmosis**

| Covariate* | Mean | 95% BCI | |
| --- | --- | --- | --- |
|  |  | 2.5% | 97.5% |
| NDVI | 0.034 | -0.014 | 0.082 |
| Distance from sea | -0.612 | -1.435 | 0.210 |
| Maximum temperature | 0.639 | -0.002 | 1.280 |
| Minimum temperature | -0.916 | -2.003 | 0.169 |
| Spatial range (km) | 21.85 | 11.005 | 39.245 |

* All covariates mean-centered and squared

Supplementary Information References

1. Priest JW, Moss DM, Visvesvara GS, Jones CC, Li A, Isaac-Renton JL. Multiplex assay detection of immunoglobulin G antibodies that recognize Giardia intestinalis and Cryptosporidium parvum antigens. Clin Vaccine Immunol. 2010;17(11):1695-707.

2. Priest JW, Moss DM, Arnold BF, Hamlin K, Jones CC, Lammie PJ. Seroepidemiology of Toxoplasma in a coastal region of Haiti: multiplex bead assay detection of immunoglobulin G antibodies that recognize the SAG2A antigen. Epidemiol Infect. 2015;143(3):618-30.

3. Rigsby P, Rijpkema S, Guy EC, Francis J, Das RG. Evaluation of a candidate international standard preparation for human anti-Toxoplasma immunoglobulin G. J Clin Microbiol. 2004;42(11):5133-8.

4. Rascoe LN, Price C, Shin SH, McAuliffe I, Priest JW, Handali S. Development of Ss-NIE-1 recombinant antigen based assays for immunodiagnosis of strongyloidiasis. PLoS Negl Trop Dis. 2015;9(4):e0003694.

5. Goodhew EB, Priest JW, Moss DM, Zhong G, Munoz B, Mkocha H, et al. CT694 and pgp3 as serological tools for monitoring trachoma programs. PLoS Negl Trop Dis. 2012;6(11):e1873.

6. Hamlin KL, Moss DM, Priest JW, Roberts J, Kubofcik J, Gass K, et al. Longitudinal monitoring of the development of antifilarial antibodies and acquisition of Wuchereria bancrofti in a highly endemic area of Haiti. PLoS Negl Trop Dis. 2012;6(12):e1941.

7. Moss DM, Priest JW, Boyd A, Weinkopff T, Kucerova Z, Beach MJ, et al. Multiplex bead assay for serum samples from children in Haiti enrolled in a drug study for the treatment of lymphatic filariasis. Am J Trop Med Hyg. 2011;85(2):229-37.

8. Priest JW, Jenks MH, Moss DM, Mao B, Buth S, Wannemuehler K, et al. Integration of Multiplex Bead Assays for Parasitic Diseases into a National, Population-Based Serosurvey of Women 15-39 Years of Age in Cambodia. PLoS Negl Trop Dis. 2016;10(5):e0004699.

9. Cooley GM, Mitja O, Goodhew B, Pillay A, Lammie PJ, Castro A, et al. Evaluation of Multiplex-Based Antibody Testing for Use in Large-Scale Surveillance for Yaws: a Comparative Study. J Clin Microbiol. 2016;54(5):1321-5.

10. Land Processes Distributed Active Archive Center (LP DAAC), Advanced Spaceborne Thermal Emission and Reflection Radiometer Global Digital Elevation Model (ASTER GDEM) Version 2. 2015, NASA EOSDIS Land Processes DAAC, USGS Earth Resources Observatoin and Science (EROS) Center: Sioux Falls, South Dakota. <http://gdem.ersdac.jspacesystems.or.jp/>.

11. Land Processes Distributed Active Archive Center (LP DAAC), Landsat 8 Operational Land Imager. 2015, NASA EOSDIS Land Processes DAAC, USGS Earth Resources Observation and Science (EROS) Center: Sioux Falls, South Dakota. Available from: <http://landsat.usgs.gov//index.php>.

12. Fornace KM, Brock PM, Abidin TR, Grignard L, Herman LS, Chua TH, et al. Environmental risk factors and exposure to the zoonotic malaria parasite Plasmodium knowlesi across northern Sabah, Malaysia: a population-based cross-sectional survey. Lancet Planet Health. 2019;3(4):e179-e86.

13. Lloyd CT, Sorichetta A, Tatem AJ. High resolution global gridded data for use in population studies. Sci Data. 2017;4:170001.

14. Fick SE, Hijmans RJ. Worldclim 2: New 1-km spatial resolution climate surfaces for global land areas. International Journal of Climatology. 2017.
