# Supplementary figures and images for: Assessing seroprevalence and associated risk factors for multiple infectious diseases in Sabah, Malaysia using serological multiplex bead assays
Source: Front Public Health. 2022 Oct 25;10:924316. doi: 10.3389/fpubh.2022.924316 (PMC9641279; doi:10.3389/fpubh.2022.924316)

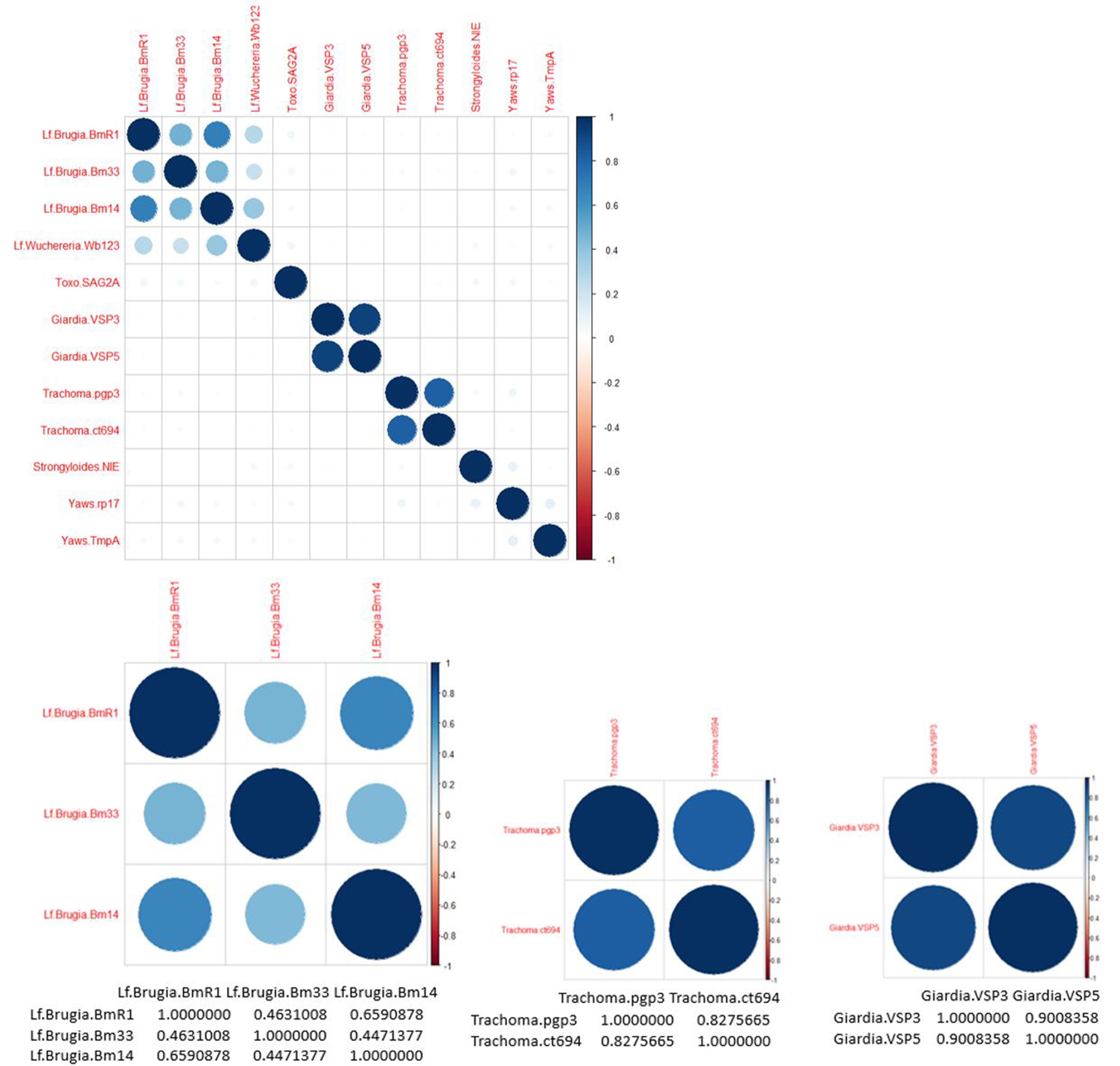

Supplement: Supplementary file 2 [file Image_1.JPEG]

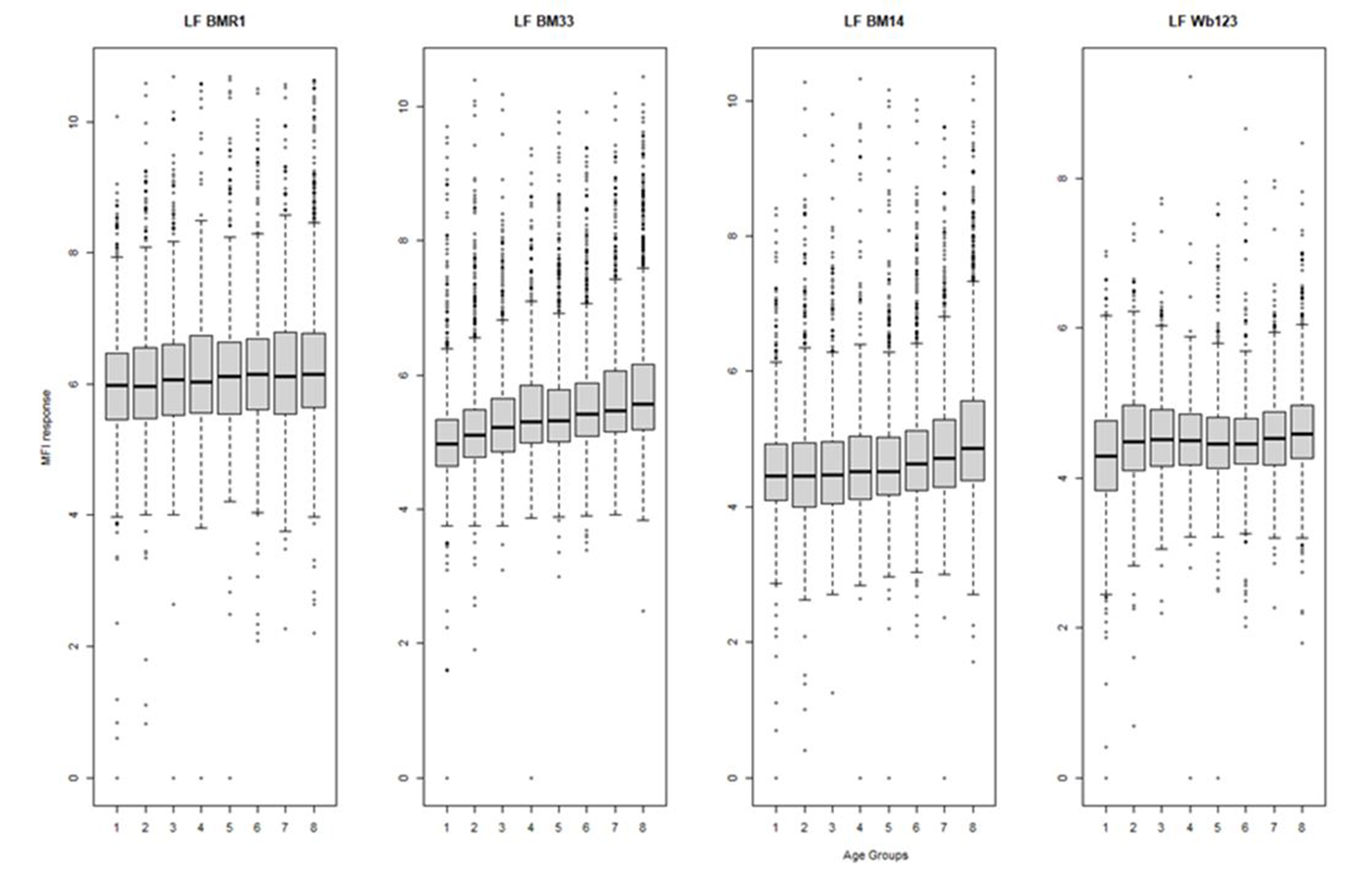

Supplement: Supplementary file 3 [file Image_2.JPEG]

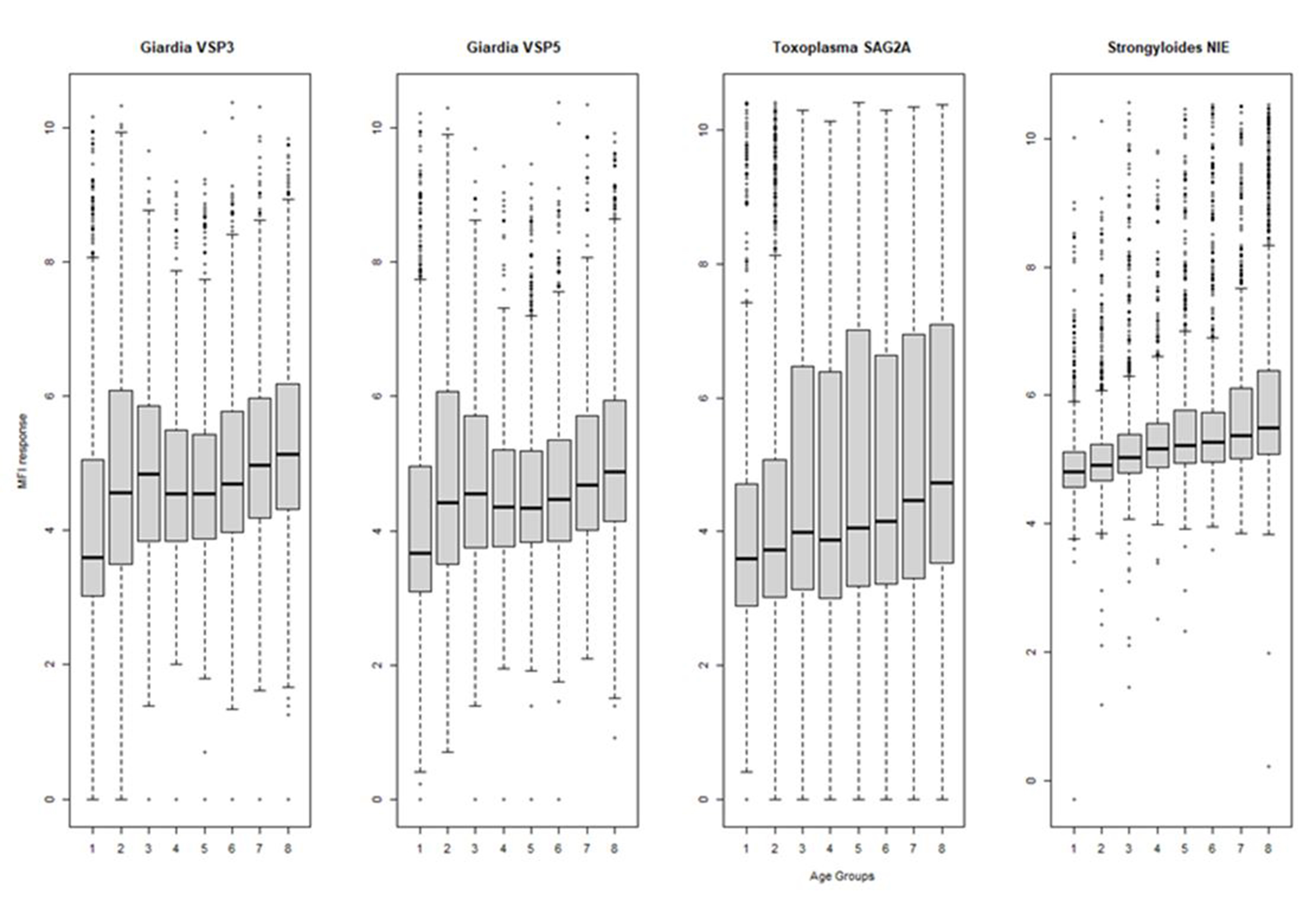

Supplement: Supplementary file 4 [file Image_3.JPEG]

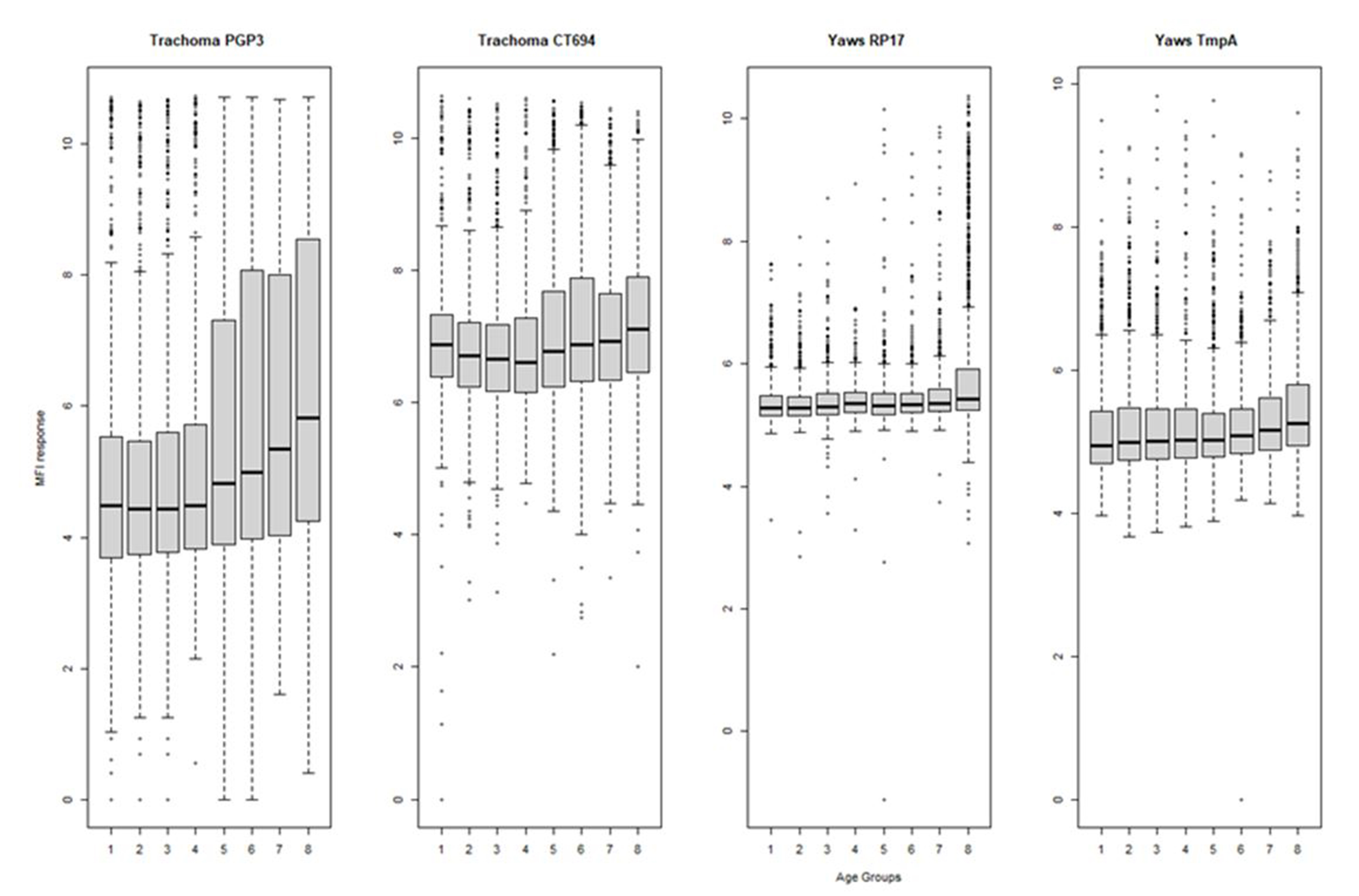

Supplement: Supplementary file 5 [file Image_4.JPEG]

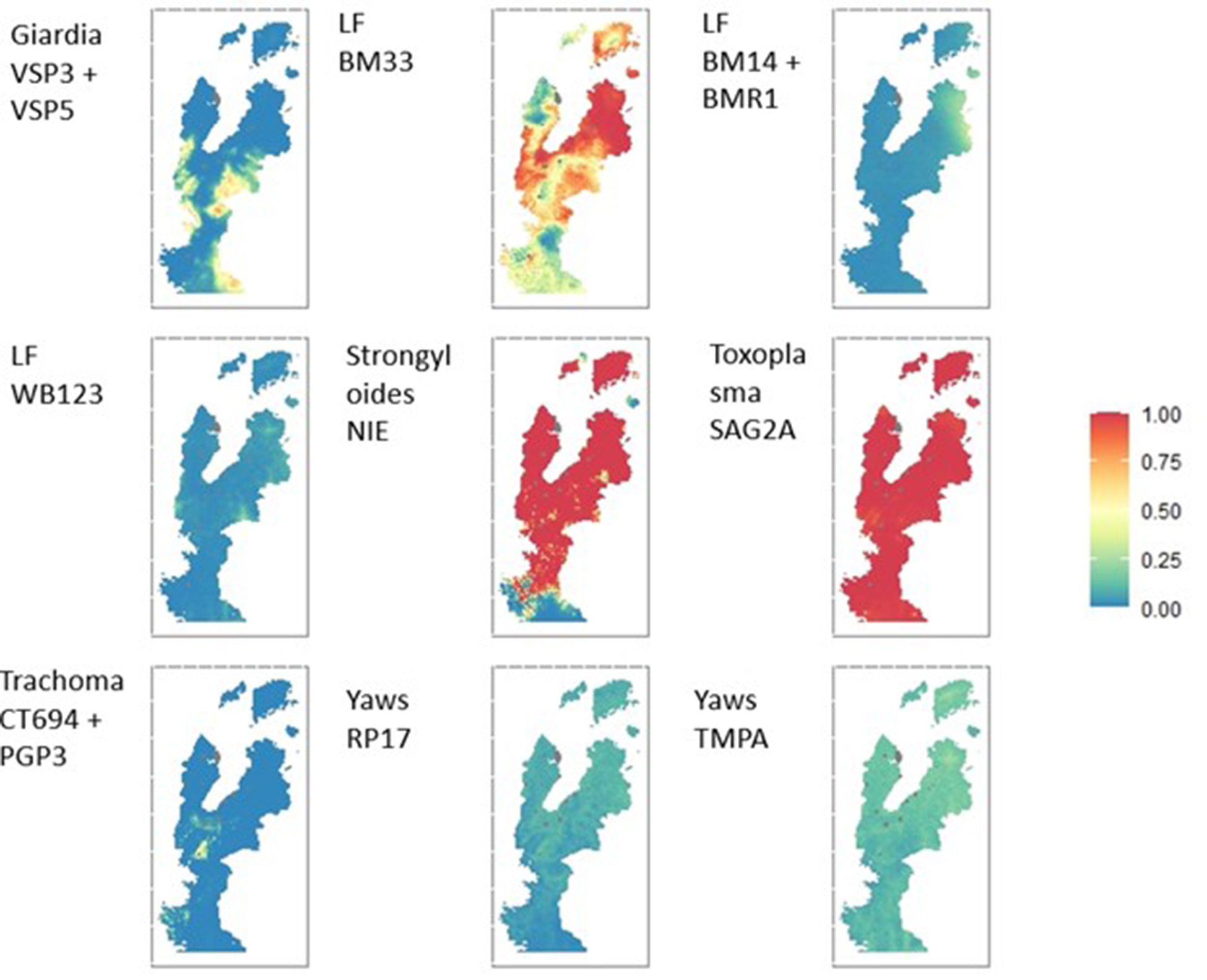

Supplement: Supplementary file 6 [file Image_5.JPEG]

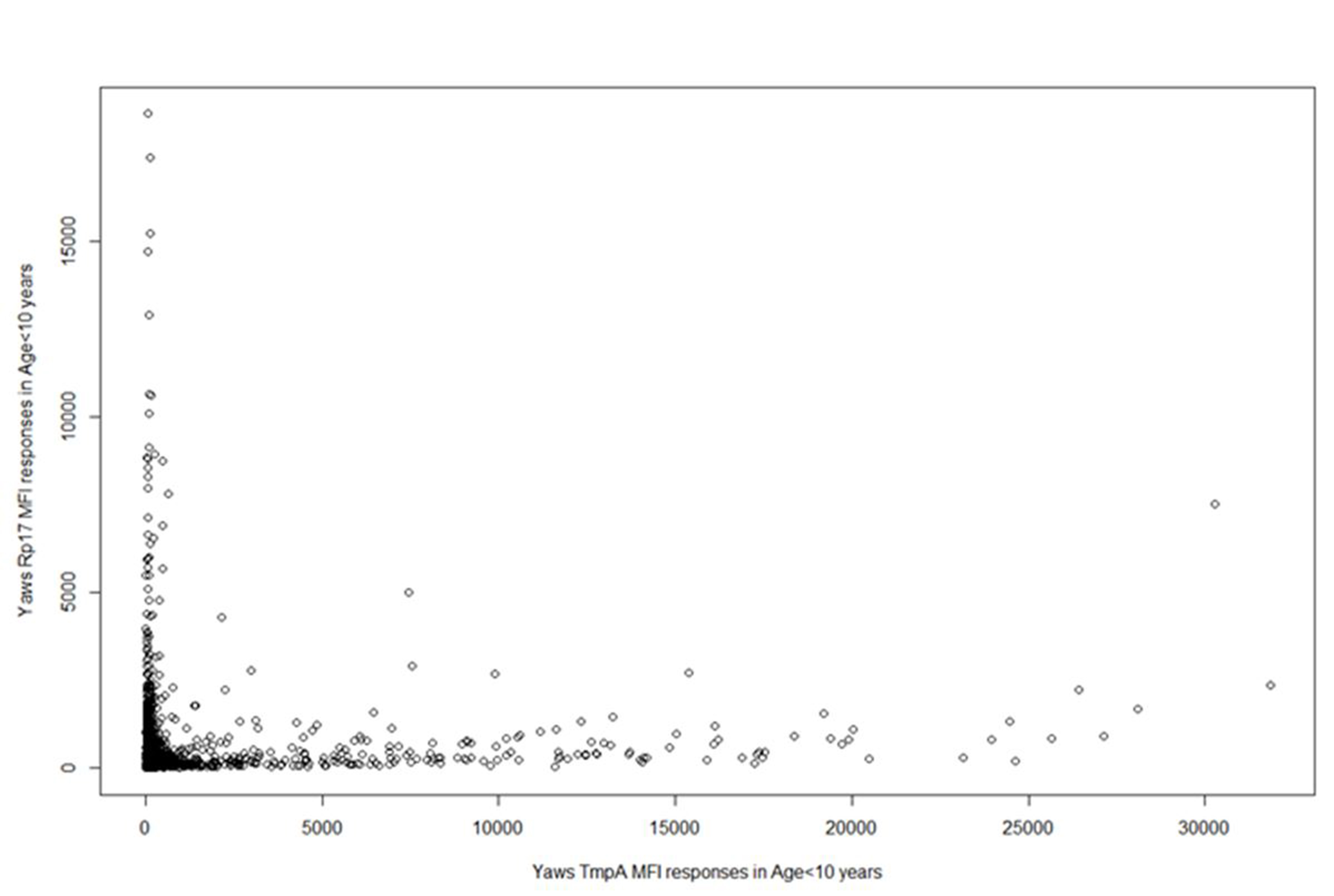

Supplement: Supplementary file 7 [file Image_6.JPEG]
